# Intra-Placental Uterine Rupture

**Published:** 1915-10

**Authors:** 


**Intra-Placental Uterine Rupture.** J. M. Stoddard, Anderson, Ind., Jour. of Ind. State Assn., September, quotes Jolly's statistics of 230 cases of rupture of uterus in 782,741 labors. Complete rupture through the placental site is one of the rare varieties of this rare condition. The patient was a primipara, aged 23, well until marriage, but had had 6 abortions at 2 or 3 months and an attack of typhoid since then. Change in the character of pains, and distention of abdomen with fluid led to diagnosis. Operation was performed in extremis but death occurred.

